# A Moderated Mediation Model of Wellbeing and Competitive Anxiety in Male Marathon Runners

**DOI:** 10.3389/fpsyg.2022.800024

**Published:** 2022-04-01

**Authors:** Jose C. Jaenes, David Alarcón, Manuel Trujillo, María del Pilar Méndez-Sánchez, Patxi León-Guereño, Dominika Wilczyńska

**Affiliations:** ^1^Department of Social Anthropology, Basic Psychology and Public Health, Pablo de Olavide University, Seville, Spain; ^2^Grossman School of Medicine, New York University, New York, NY, United States; ^3^Faculty of Higher Studies Zaragoza, National Autonomous University of Mexico, Mexico City, Mexico; ^4^Health, Physical Activity and Sports Science Laboratory (HealthPASS), Department of Physical Activity and Sports, Faculty of Education and Sports, University of Deusto, Bilbao, Spain; ^5^Department of Social Science, Gdańsk University of Physical Education and Sport, Gdańsk, Poland

**Keywords:** positive psychology, emotions, sports, self-confidence, quality of life, motivation

## Abstract

Running marathons is an increasingly popular activity with an ever-increasing number of events and participants. Many participants declare that they pursue a variety of goals by running, namely, the maintenance of good health, the development of strength and improvement of fitness, the management of emotions, and the achievement of resilience and psychological wellbeing (PWB). The research has examined marathon running, like many other sports, and has studied various factors that reduce athletic performance, such as the experience of anxiety, and that enhance such performance, such as an increase in general wellbeing. This article reports the results of a study on the experience of competitive anxiety among 238 male marathon runners who participated in Seville’s (Spain) 26th Marathon race on February 23, 2020, and investigates the relationship between anxiety and key dimensions of wellbeing as measured by the Spanish-adapted 20-item PWB Scale. We hypothesized that participating athletes who rated high on the dimensions of PWB would experience lower levels of competitive anxiety with respect to this race. We also proposed that PWB would function as a mediating factor with respect to the experience of anxiety. The results show, as hypothesized, that marathon running enhances wellbeing and reduces anxiety. The data showed significant negative correlations between four of five wellbeing dimensions and the three types of anxiety measured, namely, somatic anxiety, worry, and concentration-impairing anxiety. Other findings supported our hypothesis that wellbeing, as measured, functions as a mediating factor for the moderation of competitive anxiety. Generalization of these findings is limited by the fact that the low number of female participants recruited did not permit valid statistical analyses in this respect. It is known that both anxiety and wellbeing are subjects to variation by gender. The future inclusion of male and female subjects in equivalent studies will undoubtedly add valuable information concerning the dynamics of anxiety and wellbeing. The implications of these findings and the limitations of the study will be discussed.

## Introduction

### The Mental Health of Marathon Runners: Anxiety

At present, marathon running is one of the most popular sports activities worldwide (Vitti et al., 2020). The number of participants in marathon events has increased dramatically, with runners of many nationalities, different categories of competition, and a broad age range competing in the same event (García-Vallejo et al., 2020). One such example is the Seville Marathon, held in Seville, Spain, which is the flattest marathon course in Europe; 14,039 runners participated in this marathon in 2020 according to official statistics (Deporticket, 2022).

Performance in marathon running has been found to be influenced by various factors (Sjödin and Svedenhag, 1985) related to the complex biological adaptations that organisms must undergo in order to compete with respect to such an intense biological and psychological challenge. Such adaptations are needed to counter biological features, as exercise-induced fatigue and optimal locomotor energy utilization through self-pacing (Sperlich, 2016), and to regulate emotional experiences to deal with the stimulus overload often associated with running long distances (Jaenes and Caracuel, 2016).

Precompetitive anxiety, which is reported to be one of the most common emotions experienced among athletes in high-performance sports, affects cognitive, physiological, and locomotor skills. Such anxiety can have both positive and negative effects, which makes the ability to manage this anxiety as one of the most important parts of an athlete’s preparation (Muñoz et al., 2017). Numerous previous studies have reported that excess anxiety is one of the most challenging factors that athletes face before running a marathon. In a sample of more than 1,500 marathon runners, most runners who improved their best running time had moderate prerace scores in cognitive anxiety, very low scores in somatic anxiety, and moderate-to-high scores in self-confidence (Jaenes, 2001; Jaenes and Caracuel, 2005, 2016). While a baseline of moderate cognitive anxiety may help motivate the runner, debilitating somatic anxiety must be prevented if the athlete is to achieve a good performance. Other findings support the hypothesis that self-confidence-derived wellbeing reduces competitive anxiety among athletes (Tracey and Elcombe, 2004; Houltberg et al., 2018).

### The Mental Health of Marathon Runners Beyond Episodic Symptoms: Self-Confidence

Self-confidence, understood as the belief that one can successfully engage in a desired behavior (Otten, 2009; Weinberg and Gould, 2011), is considered to be an influential factor in athletic performance, and it has been found that the higher the levels of self-confidence in an athlete are, the lower the levels of cognitive and somatic anxiety the athlete experiences before a competition (Bejek and Hagtvet, 1996). Jones et al. (1993) also found that self-confidence has a positive effect on athletic performance. Bandura (1997) and Woodman and Hardy (2003) highlighted self-confidence as the best predictor of athletic performance.

For marathon runners, self-confidence tends to grow with experience. Studies developed by Jaenes and Caracuel (2016) have shown that runners who have completed a few marathon races experience lower levels of cognitive and somatic anxiety and report higher levels of self-confidence than less-experienced runners. Perhaps repeated running of marathons acts in these cases similar to the “exposure condition” in clinical behavior therapies, i.e., by reducing novice runners’ anxiety and enhancing their self-confidence. Jaenes and Caracuel (2016) also found a relationship between the age and the expression of anxiety. Marathon runners who aimed to improve their best time experienced medium to high levels of cognitive anxiety, low levels of somatic anxiety, and high levels of self-confidence. Effectively, these runners improved their “best time ever” in such races through their substantial competitive drive, a drive that fits the individualistic ethos ascribed to them by sports investigators (Raglin and Hanin, 2000).

It is also known that prerace levels of anxiety can be moderated by psychological interventions if anxiety becomes overwhelming or disorganizing. Jaenes et al. (2021) demonstrated the effectiveness of a psychological intervention based on the Psychological Skill Training program (PST) to help amateur male marathon runners cope with such excess anxiety and the attendant disturbing and impairing negative thoughts that could affect runners’ performance.

### The Mental Health of Marathon Runners Beyond Episodic Symptoms: Self-Esteem and Self-Efficacy

Self-efficacy refers to personal judgments and the ongoing assessment of one’s capability to organize and implement successful behaviors in specific situations. People gain information regarding their level of self-efficacy from self-performance, vicarious experience, verbal persuasion, and physiological indices. In forming judgments regarding efficacy, people consider the factors, such as perceived ability, task difficulty, effort expenditure, performance aids, and outcome patterns. Even when people acquire efficacy information from self-performance, efficacy judgments are not mere reflections of those performances. Anstiss et al. (2020) conducted a qualitative, structured interview study of athletes and found that athletes make use of several sources of self-efficacy in the formation and maintenance of self-efficacy beliefs. Specifically, the culmination of experiences, repeated experiences of overcoming challenges and adversity, and a sense of physical familiarity appeared to be key sources in the context of endurance sports.

Previous studies have shown that self-efficacy is not only an important component of sports success (Moritz et al., 2000), but also has a positive protective effect on athletes’ mental health (Treasure et al., 1996), depression, and health promotion (Bandura, 2004). This study aims to explore whether runners’ competitive anxiety is modified in the context of a positive psychological paradigm: Ryff’s dimensions of wellbeing. As the mental health field, broadly speaking, shifts from a focus on illness to a focus on health, wellbeing, and performance outcomes are enriched by a dialectic between symptoms (e.g., anxiety, depression, and psychosomatic illness) and personal characteristics, such as self-confidence, self-esteem, and self-efficacy that involve the person (self) as a whole. Such an approach, if successful, can bring about the development of new evidence-based interventions for the treatment of symptoms and the promotion of health.

There are many reasons to study the paradigm of psychological wellbeing (PWB) in the context of athletes in general and that of marathon runners in particular. In fact, many athletes have reported that they participate in sports due to the reasons, such as the opportunity to achieve self-sufficiency and independence, the ability to experience pride in their accomplishments, and the opportunity to enhance their personal growth and development (Ogles and Masters, 2003; León-Guereño et al., 2020), which are implicit dimensions of wellbeing that are consistent with Carol Ryff’s model of PWB (Ryff, 1989).

### Wellbeing in Athletes: Hedonic and Eudaimonic Perspectives

Similarly, Lundqvist (2011) studied wellbeing in sports and described two main theoretical foundations for wellbeing, namely, hedonic wellbeing and eudaimonic wellbeing. Hedonic wellbeing derives from an awareness of “feeling good,” while eudaimonic wellbeing results from a belief that one is “functioning well.” While hedonic wellbeing includes personal “subjective wellbeing” (the level of positive and negative emotions experienced by the runner combined with personal “life satisfaction” in sports), eudaimonic wellbeing focuses on the experience of functional capacity and social mastery; both domains are recognized to be important for human psychological and social growth and development. Together, these factors may provide a transcendent sense that adds richness to life. Lundqvist’s description of PWB in sports was based on Carol Ryff’s model of PWB (Ryff, 1989; Lundqvist, 2011). Ryff proposed six dimensions of PWB, namely, self-acceptance (e.g., awareness and embrace of one’s personal strengths and limitations), personal growth (e.g., a sense of the continued development of one’s potential), purpose in life (e.g., beliefs and goals that make life meaningful), autonomy (e.g., a sense of self-determination, independence, personal agency, and self-regulation), environmental mastery (e.g., the capacity of being effective with respect to managing situations in life), and positive relations with others (e.g., having quality interpersonal relationships with valued others). Ryff underlined that her concept’s strength lies in the fact that it treats PWB holistically, viewing it as an inseparable element of proper human development (Ryff, 2008, 2014; Ferguson et al., 2014, 2015; Karaś, 2017; Kouali et al., 2018).

This study focuses on variables that are equivalent to the dimensions of eudaimonic wellbeing, at the core of which lies self-determination theory (SDT) (Deci and Ryan, 1985); this theory maintains that there is a drive that prompts athletes to choose their behavior and actions with the goal of satisfying basic psychological needs (i.e., autonomy, competence, and relatedness). Such needs are essential for optimal development, personal integrity, and wellbeing in the context of sports (Ryan and Deci, 2000; Kouali et al., 2018). Wellbeing researchers have found that athletes with a high level of wellbeing enjoy greater positivity, persevere in goal-directed efforts, and adopt proactive responses to difficulties encountered in life and sports. Jordalen and Lemyre (2015), in a longitudinal study of marathon runners, found that satisfaction of the needs for autonomy and competence had the greatest influence on wellbeing over the course of a 2-month training period.

Considered from a wider perspective, wellbeing is an indicator of the healthy function of an organism, which facilitates experiences of vitality, psychological flexibility, and a deep inner sense of contentment (Ryan and Frederick, 1997). In a sample of 477 recreational marathon runners (Tian et al., 2020), serious leisure (Stebbins, 1982) was positively associated with leisure satisfaction and subjective wellbeing. Other studies have confirmed that such serious leisure qualities are also positively associated with persistent marathon running efforts, often over the course of years, in which athletes maintain the required frequency and duration of marathon events (Qiu et al., 2019). Wellbeing parameters, especially among marathon runners, seem to be very important for preventing the reported adverse physical and psychological effects of participating in elite extreme ultramarathon running (Brice et al., 2020).

### Study Aims

This study aims to investigate, among voluntary participants who are dedicated marathon runners about to compete in an important race, the relationship between PWB as defined by Ryff (and measured through a questionnaire based on Ryff’s views) and the presence of competitive anxiety. We proposed that athletes who rate high on PWB experience lower levels of competitive anxiety and that the relationship between anxiety and PWB domains exerts a meditating or moderating effect on the experience of anxiety with respect to this competitive marathon race.

Certain studies have shown crucial differences in the psychological factors that motivate marathon runners between men and women (Ogles and Masters, 2003). Achievement and competition motivation were more associated with male runners, while female runners were more motivated by lifestyle or the enthusiasm generated by running. Similarly, Larumbe-Zabala et al. (2019), in the context of a sample of Spanish marathon runners, found that women showed differences compared to their male counterparts in certain psychological variables despite exhibiting a similar relative performance level. Men scored higher in self-confidence, whereas women reported higher cognitive and somatic anxiety. Moreover, recent studies have shown that these psychological differences due to gender among marathon runners, in turn, may affect their performance during the race (Deaner et al., 2015; Nikolaidis et al., 2018; Cuk et al., 2020). In particular, men are more likely to start the marathon faster and then lower their pace, while women have less variable pacing throughout the race.

Given the previously reported differences in psychological factors among marathon runners according to gender and the proven differences between men and women with respect to marathon performance (for a review, see Casado et al., 2021), this study, due to the difficulty of obtaining a representative sample of women, addresses running experience and the wellbeing dimensions associated with precompetitive anxiety in male marathon runners.

## Materials and Methods

### Samples and Procedures

The study sample consisted of 238 marathon adult male long-distance runners aged 18–65 years (*M* = 41.22; *SD* = 8.66). The mean best time in marathon running was *M* = 12,382 s (3 h 26 min 22 s) and *SD* = 2,032 (0 h 33 min 52 s). Eighty-six percent of participants had completed at least one previous marathon in their lives (*M* = 5.93, *SD* = 8.54). Runners were voluntary subjects recruited from participants in the 26th Seville’s International Marathon, which took place in Seville, Spain (February 23, 2020). After providing informed consent, study athletes volunteered and completed the assessment questionnaires 24 h before the initiation of the marathon race. All subjects were advised that all data obtained for the study would be protected, processed anonymously, and used only for research purposes.

### Recruitment

All participants were recruited from the Seville Marathon Expo, a site that runners must visit in person directly prior to the race to collect their race material. Ideally, the study would have included an adequate sample of female runners, and we attempted to recruit such a sample. Participation was offered to men and women in the manner discussed below. Unfortunately, we were able to recruit only 16 female participants, and even though our ratio of female recruits, at 6.7% (16/238), was higher than the ratio of female recruits in the population of all 26th Seville marathon runners (3% as reported by the organizers), it was not of sufficient size to permit valid statistical inferences. Recreational runners voluntarily participated in the questionnaire collection process. The organizers of the marathon provided a stand at the Expo under the name of Pablo de Olavide University and Andalusian Center of Sports Medicine (CAMD) and informed runners by the public address of the possibility of volunteering for a marathon study, but they did not offer an endorsement. Interested subjects met with one of the three study psychologists present at the event, who informed the runners of the study characteristics and obtained signed informed consent. No names were registered. Registration completion times were between 4 and 8 min. Inclusion criteria included Spanish nationality, competing at the national or international level, and being officially registered in the Sevilla Marathon. Participation was offered to all runners, i.e., men and women, who presented themselves at our recruitment stand, which was located close to the registration station and was easily visible and accessible. Unfortunately, as explained earlier, only 16 women completed the participation documents and signed consent forms. This number was far too limited to permit meaningful statistical evaluation.

### Ethics Statement

The study was approved by the CAMD Project CAMD-PSY2020/01, operating under the guidance of the Andalusian Education and Sports Council and the Organization of the International Sevilla Marathon, and was conducted in accordance with the Declaration of Helsinki. Participants could withdraw from the survey at any time without providing justification.

### Measures

#### Competitive Anxiety

Competitive anxiety was assessed through the Spanish-adapted Sport Anxiety Scale-2 (SAS-2), a widely used measure of competitive trait anxiety experienced by athletes immediately before a competition (Ramis et al., 2010). The SAS-2 (Smith et al., 2006) is a 15-item questionnaire that assesses the competitive trait anxiety experienced by athletes before or during competition. The SAS-2 measures responses for three anxiety subfactors, namely, somatic anxiety, worry, and concentration-impairing anxiety. Participants rated each item on a four-point Likert-type scale ranging from one (not at all) to four (very much). The Cronbach’s alpha coefficients for the three subscales ranged from 0.67 to 0.83 for this study. Although the original and Spanish-adapted versions were validated mainly in the context of children and young adults (Smith et al., 2006; Ramis et al., 2010, 2015), recent studies have tested the validity of the SAS-2 with respect to older adults (Coreia et al., 2017; Cho et al., 2018; Silva-Rocha et al., 2019). For instance, Madrigal et al. (2018), in the context of a sample of 542 athletes with an age range from 18 to 72 years, confirmed the factor structure of the scale in three dimensions.

#### Psychological Wellbeing

The Spanish-adapted reduced 20-item PWB Scale (Díaz et al., 2006) was employed to assess six aspects of wellbeing, namely, autonomy (e.g., “I have confidence in my opinions, even if they are contrary to the general consensus”), environmental mastery (e.g., “In general, I feel that I am in charge of the situation in which I live”), personal growth (e.g., “I think that it is important to have new experiences that challenge how you think about yourself and the world”), positive relationships with others (e.g., “People would describe me as a giving person who is willing to share my time with others”), purpose in life (e.g., “Some people wander aimlessly through life, but I am not one of them”), and self-acceptance (e.g., “When I look at the story of my life, I am pleased with how things have turned out”). Participants rated each item on a six-point Likert-type scale ranging from one (strongly disagree) to six (strongly agree). The Cronbach’s alpha coefficients for the six subscales ranged from 0.55 to 0.85 for this study.

The scale has been extensively used to study wellbeing in large general population studies, such as the US National Survey of Families and Households (Sweet and Bumpass, 1996), the Midlife in the United States (Brim et al., 2004), the Canadian Study of Health and Aging (Clarke et al., 2001), and the English Longitudinal Study of Aging (ELSA). The Ryff’s items have also been used as wellbeing outcome indicators in smaller surveys, such as studies of body consciousness (McKinley, 1999), life challenges (McGregor and Little, 1998) or midlife work aspirations (Carr, 1997), and studies concerning the outcomes of therapeutic interventions (Fava et al., 2005).

### Data Analysis

All statistical procedures were conducted using IBM SPSS Statistics version 25. In descriptive statistics, although there is no consensus regarding marathon age categories by following previous studies of marathon-running populations (Jokl et al., 2004; Lepers and Cattagni, 2012; León-Guereño et al., 2021), our participants were classified into the following groups: young adults (18–29 years), middle adults (30–40 years), veteran adults (40–50 years), and senior adults (50–65 years). The supposition of normality was checked using the Kolmogorov-Smirnov test, and the results found a non-normal distribution of variables with respect to the number of marathons completed and for the wellbeing and competitive anxiety measures. Subsequent to that finding, the non-parametric Kruskal-Wallis test (K-W) was used to account for differences by age range. The Spearman’s rank correlation tests were performed to analyze the bivariate correlations among the wellbeing and competitive anxiety dimensions. Multiple linear regression analysis was performed using each anxiety dimension as a dependent variable. Possible predictors considered included age in years, number of marathons completed, rank of the best mark in seconds, and wellbeing dimensions. The supposition of the independence of residuals was verified *via* the Durbin-Watson statistic, and the normality of their distribution justified the use of the analysis despite the non-normality of the variables. The bilateral level of significance established prior to all the tests was 0.05. The PROCESS macro version 3.5 for SPSS (Model 5; Hayes, 2013) was used to determine the mediating and moderating effects of marathon experience on competitive anxiety. For this PROCESS model, a bootstrapping procedure was selected with 10,000 bootstrap samples used to calculate bias-corrected 95% CI, which were robust against violations of the normality assumption. A significant effect was considered to exist if the CIs did not include zero.

## Results

### Descriptive Statistics

#### Race Performance or Running Experience by Age

A non-parametric K-W was performed to determine whether there were any significant differences in the number of marathons completed or the best time achieved in a marathon as a function of the age range of the athletes. Table 1 shows the mean (SD) of the number of marathons completed and the best time in seconds by athletes’ age range. A significant difference by age range was obtained for the number of marathons completed (K-W = 30.513, *p* < 0.01). As expected, older athletes had completed a greater number of marathons in the past. However, no significant differences by age range were found in the best mark obtained in a marathon (K-W = 2.635, *p* > 0.05). Thus, “performance” does not seem to be determined by age or experience.

**TABLE 1:** Descriptive by age ranges.

	Age				
	
	18–30 *n* = 26 *M* (*SD*)	31–40 *n* = 89 *M* (*SD*)	41–50 *n* = 92 *M* (*SD*)	51–65 *n* = 31 *M* (*SD*)	Kruskal-Wallis
Number of marathons finished	3.04 (4.46)	3.72 (4.27)	6.45 (6.61)	14.77 (13.17)	30.513***
Best-mark	13385.66 (3590.05)	12206.45 (1685.81)	12382.11 (1871.45)	12158.17 (1910.80)	2.635
Wellbeing	4.66 (0.65)	4.71 (0.68)	4.80 (0.61)	4.97 (0.68)	5.547
Self-acceptance	4.95 (0.80)	4.90 (0.85)	4.97 (0.83)	5.17 (0.86)	3.850
Autonomy	3.79*^a^* (0.47)	3.82*^a^* (0.67)	4.00 (0.71)*^a^*	4.28*^b^* (0.46)	16.202**
Environmental mastery	4.70 (0.96)	4.74 (0.84)	4.80 (0.72)	5.01 (0.83)	2.916
Purpose in life	4.70 (0.89)	4.82 (1.01)	4.92 (0.84)	5.10 (0.87)	3.822
Personal growth	5.08 (0.78)	5.09 (0.82)	5.12 (0.88)	5.28 (0.83)	2.305
Positive relations	4.74 (1.14)	4.92 (0.92)	4.99 (0.87)	4.99 (1.02)	0.927
Anxiety	1.74 (0.40)	1.69 (0.45)	1.67 (0.37)	1.53 (0.32)	5.548
Somatic	1.77 (0.41)	1.80 (0.56)	1.72 (0.47)	1.57 (0.33)	4.563
Worry	2.12 (0.72)	1.89 (0.64)	1.92 (0.62)	1.75 (0.54)	4.281
Concentration Disruption	1.34 (0.33)	1.39 (0.46)	1.38 (0.37)	1.27 (0.37)	3.470

**p < 0.01; ***p < 0.001. Letters show group significant differences by age.

#### Wellbeing Dimensions by Age

The non-parametric K-W tests were conducted to analyze differences in the wellbeing and anxiety dimensions of the athletes according to age range. Table 1 shows the means (*SD*) of the wellbeing and anxiety dimensions divided by athletes’ age ranges. A significant difference by age range was obtained only for the autonomy dimension of the wellbeing scale (K-W = 16.202, *p* < 0.01). A *post hoc* Bonferroni corrected Mann-Whitney *U*-test showed that adults older than 50 years had higher premarathon autonomy scores than did adult groups aged 50 years or younger (*p*-values < 0.05). Age seems to enhance pre-existing autonomy in athletes, which is the only PWB dimension affected by age.

### Correlation Analysis of Scale Variables of Anxiety and Wellbeing Subdimensions

The Spearman’s rank correlation analysis was conducted to analyze bivariate relationships among the wellbeing and anxiety scale subdimensions (refer to Table 2). According to the studies by Cohen (1988, 1992), moderate positive correlations were found between the five wellbeing dimensions (*r*s > 0.3, *p*-values < 0.001). However, a small correlation was found between personal growth and autonomy (*r* = 0.262, *p*-values < 0.001) and between positive relationship and autonomy (*r* = 0.291, *p*-values < 0.001). Similarly, the three anxiety dimensions were moderately positively correlated (*r*s > 0.3, *p*-values < 0.001). In accordance with Cohen (1988, 1992), small significant negative correlations were found among most of the wellbeing dimensions and the three anxiety types measured, namely, somatic anxiety, worry, and concentrating-impairing anxiety (*r*s < –0.2, *p*-values < 0.01). Furthermore, worry was moderately negatively correlated with self-acceptance (*r* = –0.322, *p*-values < 0.001) and autonomy (*r* = –0.301, *p*-values < 0.001). However, as described earlier, none of the anxiety dimensions were significantly correlated with the autonomous dimension of wellbeing (*p*-values > 0.05).

**TABLE 2:** The Spearman’s rank correlations.

	1	2	3	4	5	6	7	8
1. Self-acceptance	–							
2. Autonomy	0.384**	–						
3. Environmental mastery	0.636**	0.370**	–					
4. Purpose in life	0.692**	0.318**	0.620**	–				
5. Personal growth	0.655**	0.262**	0.573**	0.765**	–			
6. Positive relations	0.588**	0.291**	0.584**	0.620**	0.550**	–		
7. Somatic anxiety	–0.281**	–0.065	–0.168*	–0.223*	–0.159*	–0.159*	–	
8. Worry	–0.322**	–0.091	–0.301**	–0.233**	–0.270**	–0.259**	0.484**	–
9. Concentration disruption	–0.258**	–0.061	–0.269**	–0.246**	–0.260**	–0.265**	0.399**	0.388**

*p < 0.05; **p < 0.01.

### Regression Analysis Predictors of Anxiety Dimensions

Multiple regression analyses were conducted to analyze the wellbeing dimensions accounting for somatic anxiety, worry, and concentration-impairing anxiety, controlling for age in years, number of marathons completed, and the highest running mark ever achieved. Somatic anxiety was significantly predicted by the wellbeing component of self-acceptance (β = –0.25; *p* < 0.05; refer to Table 3). Environmental mastery (β = –0.23; *p* < 0.05) and self-acceptance (β = –0.22; *p* < 0.05) accounted significantly for the variability of competitive worry (Table 4), and environmental mastery significantly predicted concentration disruption (β = –0.29; *p* < 0.01) (Table 5). This finding is noteworthy: corresponding to high levels of self-acceptance, we found lower levels of anxiety. It is interesting to note that self-acceptance-based therapies are the leading approach in the context of therapeutic psychosocial interventions for anxiety in clinical studies among the general population. Our study shows that self-acceptance is a very significant mediator of anxiety (and a predictor of lower anxiety) in our male marathon runner subjects and agrees with findings in the literature, showing that self-acceptance-based therapies are very effective interventions in the clinical treatment of anxiety and mood disorders. If it was replicated and extended to female runners, our findings would support clinical trials of the application of self-acceptance interventions to sports anxiety. Similar comments can be made regarding the finding that enhanced environmental mastery helps reduce concentration-impairing anxiety.

**TABLE 3:** Regression analysis summary for predicting somatic anxiety.

Predictors	b	SE	*B*	T	*p*
Age in years	–0.03	0.02	–0.105	–1.453	0.148
Marathons finished	–0.006	0.025	–0.021	–0.259	0.796
Best-mark classification	0.086	0.076	0.081	1.13	0.26
Wellbeing					
Self-acceptance	–0.725	0.302	–0.249	–2.399	0.017
Autonomy	0.191	0.257	0.052	0.743	0.458
Environmental Mastery	–0.034	0.266	–0.011	–0.126	0.900
Purpose in life	–0.254	0.306	–0.095	–0.828	0.409
Personal growth	0.329	0.311	0.113	1.059	0.291
Positive relations	–0.041	0.224	–0.016	–0.184	0.854

*R^2^ = 0.097, F(9, 237) = 2.729, p < 0.01.*

**TABLE 4:** Regression analysis summary for predicting worry.

Predictors	b	SE	β	T	*p*
Age in years	–0.039	0.026	–0.105	–1.496	0.136
Marathons finished	–0.019	0.032	–0.046	–0.591	0.555
Best-mark classification	0.023	0.096	0.017	0.236	0.813
Wellbeing					
Self-acceptance	–0.866	0.382	–0.23	–2.268	0.024
Autonomy	0.349	0.324	0.073	1.075	0.283
Environmental mastery	–0.845	0.335	–0.217	–2.521	0.012
Purpose in life	0.353	0.387	0.103	0.913	0.362
Personal growth	0.013	0.392	0.003	0.033	0.974
Positive relations	–0.122	0.282	–0.036	–0.432	0.666

*R^2^ = 0.138, F(9, 237) = 4.065, p < 0.001.*

**TABLE 5:** Regression analysis summary for predicting concentration disruption.

Predictors	b	SE	β	t	*p*
Age in years	–0.003	0.017	–0.014	–0.2	0.842
Marathons finished	0.003	0.02	0.011	0.141	0.888
Best-mark classification	0.103	0.062	0.118	1.657	0.099
Wellbeing					
Self-acceptance	0.008	0.246	0.003	0.034	0.973
Autonomy	0.273	0.209	0.09	1.308	0.192
Environmental mastery	–0.716	0.216	–0.29	–3.318	0.001
Purpose in life	0.059	0.249	0.027	0.239	0.812
Personal growth	–0.042	0.252	–0.017	–0.165	0.869
Positive relations	–0.128	0.182	–0.06	–0.706	0.481

*R^2^ = 0.109, F(9, 237) = 3.107, p < 0.01.*

### Mediation and Moderation Effect Analysis

**FIGURE 1:**
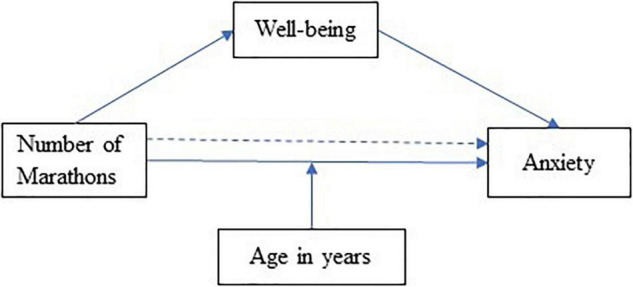
Hypothetical mediation and moderation model of the effect of the number of marathons.

**FIGURE 2:**
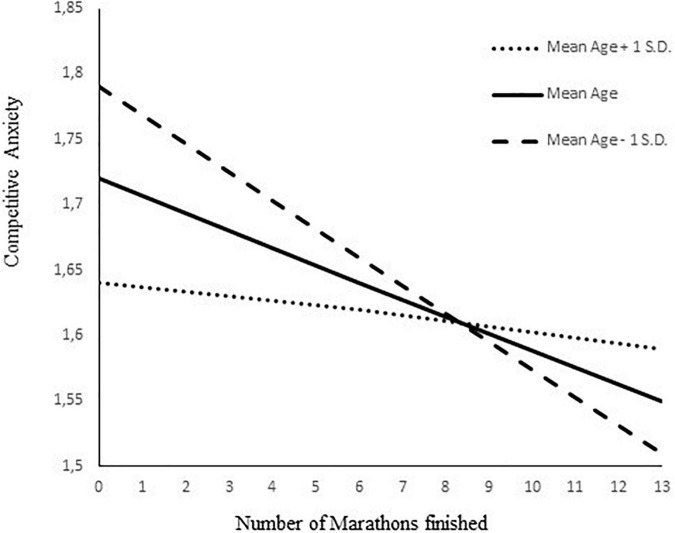
Age moderating effect between number of marathons and competitive anxiety.

To examine whether marathon experience influences wellbeing and premarathon anxiety, a moderated mediation model was tested using the number of races as the predictor, wellbeing as the mediator variable, anxiety as the dependent variable, and age in years as the moderator of the direct effect (Figure 1). The findings in Table 6 show that having completed previous marathon races increases wellbeing (β = 0.15, bootstrap 95% CI = [0.08, 0.28]) and that marathon experience reduces the level of competitive anxiety, an effect which is both directly (β = –0.23, bootstrap 95% CI = [–0.36, –0.10]) and indirectly mediated *via* wellbeing pathways (β = –0.05, bootstrap 95% CI = [–0.09, –0.02]). However, the interaction term between the number of marathons and age in years was significant (β = 0.15, bootstrap 95% CI = [0.07, 0.24]), suggesting that the direct effect of marathons completed on athletes’ experience of anxiety was moderated by athletes’ age. Table 7 shows the conditional effects on competitive anxiety of the number of marathons completed at ± 1 *SD* of the age mean. The moderation (a negative relationship) on competitive anxiety from accumulated marathon experience was stronger for younger athletes (competitive anxiety; β = –0.39, bootstrap 95% CI = [–0.65, –0.12]), intermediate for athletes at the mean age (β = –0.23, bootstrap 95% CI = [–0.41, –0.06]), and non-significant for older athletes (β = –0.08, bootstrap 95% CI = [–0.21, 0.06]). This interaction was plotted at + 1/–1 *SD* from the mean age in years (Figure 2).

**TABLE 6:** Mediation and moderation regression analysis.

				Bias-corrected 95% CI standardized	
				
	B	SE	β	Lower limit	Upper limit
**Direct effects on wellbeing**					
Marathons finished	0.0150	0.0054	0.1785	0.0816	0.2832
**Direct effects on anxiety**					
Marathons finished	–0.0508	0.0174	–0.2333	–0.3678	–0.1025
Age	–0.0085	0.0037	–0.0580	–0.1631	0.0514
Wellbeing	–0.1834	0.0380	–0.2970	–0.4291	–0.1790
Marathons finished × age	0.0009	0.0003	0.1560	0.0696	0.2385
**Indirect effect on anxiety**					
From marathons finished to anxiety *via* wellbeing	–0.0028	0.0010	–0.0530	–0.0939	–0.0218

**TABLE 7:** Conditional effects of the focal predictor (marathons completed) at ± 1 *SD* of the mean moderator (age in years).

Moderator levels				Bias-corrected 95% CI standardized	
				
Age*	b	SE	β	Lower limit	Upper limit
Mean-1 *SD* (32.54)	–0.0202	0.0069	–0.3893	–0.6500	–0.1286
Mean (41.17)	–0.0121	0.0046	–0.2333	–0.4079	–0.0588
Mean + 1 *SD* (49.79)	–0.0040	0.0035	–0.0773	–0.2114	0.0568

*Age in years values are given in parenthesis.

## Discussion

The aim of this study was to analyze the relationship between various dimensions of PWB and the presence of competitive anxiety in voluntary dedicated male marathon runners who were about to compete in an important race.

### Wellbeing Moderates Anxiety in Male Marathon Runners

The results showed that the selected dimensions of PWB negatively correlated with precompetitive anxiety dimensions, as the authors hypothesized. However, the autonomy dimension of wellbeing, as a sense of self-determination, independence, and regulation of one’s behavior, was not significantly correlated with any of the anxiety dimensions. This particular scale of wellbeing depended on the age of the marathon runners. Older athletes were characterized by a significantly higher sense of self-determination, independence, and behavior self-regulation than their younger colleagues. These wellbeing features could be related to the nature of endurance sports and the influence of engaging in the sport itself (Qiu et al., 2019; Brice et al., 2020; Tian et al., 2020). According to the study by Ryan and Deci (2007), sports demand motivation and require a great deal of self-discipline. Endurance sports consume an incredible amount of leisure time in preparation and training, and amateur athletes often treat their sport as a second job. Simultaneously, an individual’s chances of winning a competition are meager because of great variance in the abilities of participants. Nevertheless, thousands of amateur marathon runners continue to participate in sporting events, tolerating mental and physical agony with less chance of standing at the winner’s podium. These aspects require self-determination and a great deal of self-regulation, which are developed through years of practice and competition in endurance sports and have positive effects on long-term health, wellbeing, and performance (Lamont and Kennelly, 2012; Balk and Englert, 2020).

As hypothesized, significant negative correlations were found among most of the wellbeing dimensions (except for the autonomy dimension) and the three anxiety types measured, namely, somatic anxiety, worry, and concentrating-impairing anxiety. Thus, all but one of the dimensions of wellbeing measured in our subjects (e.g., self-acceptance, environmental mastery, purpose in life, personal growth, and personal relationship) are potential moderators of anxiety in male marathon runners. All these dimensions are improved by repeated marathon participation, and this fact validates many reasons that people give for participating in marathons (e.g., “to maintain good health,” “to help manage emotions,” and “to improve resilience and wellbeing”). It may be the case that autonomy has its roots in character or temperament since this trait seems to grow and develop in our subjects as a function of time (age), experience, and maturity, parameters that are relatively independent of the environment (Anstiss et al., 2020). In addition, autonomy may already be elevated in people who engage in competitive sports, such as marathon racing, and smaller gains are to be expected.

### Marathon Experience Moderates Anxiety

Similar to previous studies, we found complex relationships between anxiety during the marathon and Ryff’s six dimensions of wellbeing (Tracey and Elcombe, 2004; Houltberg et al., 2018). Premarathon anxiety is inversely correlated with having prior experience of running marathons or anxiety during the marathon. However, this accumulated experience must be translated into steady wellbeing to have a protective impact on competitive anxiety (Bejek and Hagtvet, 1996; Jaenes and Caracuel, 2016). Maturity as an athlete (as a function of age) facilitates that translation, which occurs through gains in the six dimensions of wellbeing described by Ryff.

### Maturity Moderates Anxiety, Largely *via* Wellbeing Dimensions

The analysis also revealed in the mediation model that the indirect and direct effects of wellbeing from the number of marathons completed and overall anxiety suggest a complementary (partial) mediation. Partial mediation indicates that wellbeing dimensions serve as a mediator through which an athlete’s marathon experience influences the level of overall anxiety; however, this result also suggests that wellbeing alone cannot account for all of the variances in athletes’ anxiety dimensions (Ogles and Masters, 2003). The findings of this study seem to suggest that marathoners with greater marathon experience and more number of marathons completed could experience a lower level of anxiety if their level of overall wellbeing is high (Raglin and Hanin, 2000). In contrast, the moderation model underlines the fact that the direct effect of marathons completed on anxiety was moderated by athletes’ age, and younger athletes who were less experienced at running marathons were characterized by higher competitive anxiety (León-Guereño et al., 2020).

There seems to be a synergy here, according to which the athlete gains maturity as a function of age; maturity is enhanced through the PWB gains achieved through participation in competitive racing. Reductions and increases in anxiety are mediated by the six PWB factors proposed by Ryff, namely, self-acceptance, personal growth, purpose in life, autonomy, environmental mastery, and positive relationship with others.

### Practical Implications for Holistic Training Interventions

Finishing a marathon is a tremendous achievement for marathon runners, and researchers working in the field of positive psychology underline the facts that accomplishments are an essential component of wellbeing and that people who accomplish their goals are happier (Seligman, 2012, 2004). Reaching goals with respect to marathon running is possible through years of practice, especially since endurance motor ability increases with age, and the best results are possible in early and middle adulthood; therefore, the older you are, the better you are. Pori et al. (2013), investigating Slovenian marathoners, showed that life satisfaction increased with every kilometer run within a week. Pacesova et al. (2019) proved that athletes with greater experience are characterized by significantly higher positive aspects of wellbeing, such as a more positive attitude toward life. Researchers state that physically active people are happier, are more optimistic, experience better moods, and evaluate their quality of life more highly than do inactive people (Wendel-Vos et al., 2004; Penedo and Dahn, 2005; Thøgersen-Ntoumani et al., 2005). These findings reinforce the importance of complementing the traditional focus in psychology and psychopathology on symptoms and reinforce the therapeutic value of more holistic, global, and person-centered approaches to promote health. Thus, coaches and sports clinicians can better assist athletes in overcoming the mental barriers to long-distance racing performance while utilizing selective dimensions of wellbeing supported by evidence.

In fact, there are many evidence-based interventions available in the therapeutic world, which target constructs such as self-acceptance (Hayes et al., 2006; Trujillo, 2021), purpose (Frankl, 1963; McKnight and Kashdan, 2009), personal growth (Russell, 1992), environmental mastery, and positive relationship with others. Some of these interventions have already been or can readily be adapted to help athletes in general and marathon running populations in particular. However, the questionnaire does not determine which of the dimensions measured can be conceptualized as personality traits and which must be regarded as states. Previous studies have confirmed that eudaimonic wellbeing changes after the implementation of psychological therapy (Peris et al., 2016; Colás et al., 2017; Cantón et al., 2019) or following major life events associated with life changes, such as the transition to parenthood (Brandel et al., 2018). Future research should address the question of whether a holistic training intervention might enhance wellbeing to facilitate coping with anxiety, particularly among less-experienced runners.

### Limitations

The limitations of this study include the low number of runners who completed the marathon in less than 3 h and, regrettably, the low percentage of female runners who participated in the 2020 Seville marathon and completed the research survey.

This study is also limited by certain scales used, some of which, although they have been standardized and validated, may have poor internal consistency indices. Future research must focus on a larger number of runners who complete the marathon more quickly in order to determine whether faster runners (who presumably practice more hours per week) display more significant health impacts than average or slower runners.

## Data Availability Statement

The raw data supporting the conclusions of this article will be made available by the authors, without undue reservation.

## Ethics Statement

The studies involving human participants were reviewed and approved by Andalusian Center of Sport Medicine (CAMD). The patients/participants provided their written informed consent to participate in this study.

## Author Contributions

JJ and DW conceived the study. JJ collected the data in the Sevilla Marathon and wrote the initial manuscript. DA analyzed the data, wrote the results and discussion, and provided critical revisions on the successive drafts. DW, PL-G, and MM-S provided information to complete the article and critical revisions in the successive drafts. MT provided a final critical edit, reorganized the linking and definition of variables, analyses, and findings, and additional meanings of findings, added recommendations and did a complete English revision. All authors read and approved the final version.

## Conflict of Interest

The authors declare that the research was conducted in the absence of any commercial or financial relationships that could be construed as a potential conflict of interest.

## Publisher’s Note

All claims expressed in this article are solely those of the authors and do not necessarily represent those of their affiliated organizations, or those of the publisher, the editors and the reviewers. Any product that may be evaluated in this article, or claim that may be made by its manufacturer, is not guaranteed or endorsed by the publisher.
